# Social familiarity relaxes the constraints of limited attention and enhances reproduction of group-living predatory mites

**DOI:** 10.1111/j.1600-0706.2012.20833.x

**Published:** 2013-08

**Authors:** Markus A Strodl, Peter Schausberger

**Affiliations:** M. A. Strodl and P. Schausberger (peter.schausberger@boku.ac.at), Division of Plant Protection, Dept of Crop Sciences, Univ. of Natural Resources and Life SciencesPeter Jordan Strasse 82, AT-1190 Vienna, Austria

## Abstract

In many group-living animals, within-group associations are determined by familiarity, i.e. familiar individuals, independent of genetic relatedness, preferentially associate with each other. The ultimate causes of this behaviour are poorly understood and rigorous documentation of its adaptive significance is scarce. Limited attention theory states that focusing on a given task has interrelated cognitive, behavioural and physiological costs with respect to the attention paid to other tasks. In multiple signal environments attention has thus to be shared among signals. Assuming that familiar neighbours require less attention than unfamiliar ones, associating with familiar individuals should increase the efficiency in other tasks and ultimately increase fitness. We tested this prediction in adult females of the group-living, plant-inhabiting predatory mite *Phytoseiulus persimilis.* We evaluated the influence of social familiarity on within-group association behaviour, activity, predation and reproduction. In mixed groups (familiar and unfamiliar), familiar predator females preferentially associated with each other. In pure groups (either familiar or unfamiliar), familiar predator females produced more eggs than unfamiliar females at similar predation rates. Higher egg production was correlated with lower activity levels, indicating decreased restlessness. In light of limited attention theory, we argue that the ability to discriminate between familiar and unfamiliar individuals and preferential association with familiar individuals confers a selective advantage because familiar social environments are cognitively and physiologically less taxing than unfamiliar social environments.

Group-living is a widespread phenomenon in both invertebrate and vertebrate animals (Krause and Ruxton 2002). Ultimately, animals live in groups for various reasons such as enhanced food exploitation, vigilance, protection from or defence against predators, breeding, mate choice, homeostasis or energy saving in group movements (Alexander 1974, Krause and Ruxton 2002, Earley and Dugatkin 2010). Proximately, group formation and cohesion may be based on two principal mechanisms: mutual attraction and/or response to external stimuli such as abiotic conditions or food sources (Alexander 1974, Krause and Ruxton 2002). In most cases, the proximate causes of group formation and cohesion are difficult to pinpoint because grouping is commonly guided by both conspecific and external stimuli (Krause and Ruxton 2002). While the mechanisms of group formation and cohesion, and the benefits and costs of group-living have been intensely investigated (Alexander 1974, Krause and Ruxton 2002, Earley and Dugatkin 2010), a more rarely addressed aspect of group-living is within-group association behaviour (Croft et al. 2005, Jordan et al. 2010) and its bearings for individual group members.

Within-group associations are commonly non-random and may be determined by group member characteristics such as life stage, sex, size, dominance rank or social famili arity (Conradt and Roper 2000, Krause and Ruxton 2002, Guttridge et al. 2009, Earley and Dugatkin 2010, Kaspersson et al. 2010). Here we focused on social familiarity, which requires the ability to discriminate familiar and unfamiliar conspecific individuals based on prior association (Waldman 1988, Grafen 1990, Mateo 2004). Preferential association of familiar individuals has been observed in many animal taxa such as mammals (Palphramand and White 2007), birds (Komdeur et al. 2004), fish (Höjesjö et al. 1998, Griffiths 2003), amphibians (Blaustein and O’Hara 1986) and arthropods (Gamboa 2004, Schausberger 2004, 2007, Strodl and Schausberger 2012a, b).

Familiarity is a common mechanism used to discriminate kin and non-kin (Blaustein and O’Hara 1986, Waldman 1988, Mateo 2004, Schausberger 2007). The adaptive significance of familiarity, if used as proxy of genetic relatedness, has been revealed for various group- living animals (Komdeur et al. 2004, Gamboa 2004, Schausberger 2004, 2007). If there is an obvious gain in inclusive fitness, preferential treatment of familiar individuals is commonly considered kin-selected behaviour (Hamilton 1964) and kin-selected behaviours targeted towards non-kin would be considered recognition errors. However, several studies showed that differential treatment of familiar and unfamiliar individuals may occur inde pendently of the degree of genetic relatedness and may be beneficial without indirect fitness gains, implying the existence of alternative or additional forces selecting for the ability to discriminate familiar and unfamiliar individuals. For example, familiarity of group members, independent of genetic relatedness, may increase foraging efficiency (Griffiths et al. 2004, Strodl and Schausberger 2012b) or may reduce aggressive and competitive behaviours (Ward and Hart 2003, Palphramand and White 2007, Höjesjö et al. 1998), emotional stress (Takeda et al. 2003) or predation risk (Chivers et al. 1995, Ward and Hart 2003, Strodl and Schausberger 2012a). While all these studies show the behavioural consequences of familiarity and commonly assume familiarity to be adaptive, only few studies linked the behavioural observations with the interrelated cognitive processes (Griffiths et al. 2004, Strodl and Schausberger 2012a), or provided a conclusive ultimate explanation for why the ability to recognise familiar individuals evolved in and should be beneficial for group- living animals. Moreover, only one study (Strodl and Schausberger 2012a) experimentally documented the adaptive significance, i.e. the effects of social familiarity on the prime fitness traits, survival and/or reproduction. Strodl and Schausberger (2012a) observed enhanced survival of socially familiar mites under the risk of predation as compared to unfamiliar mites.

A highly appealing, but rarely experimentally tested, ultimate explanation of the benefits of associating with familiar individuals and the interrelated cognitive processes is the implication of limited attention (Dukas 2002). Limited attention theory postulates that focusing on a given task has cognitive and associated physiological costs with respect to the attention paid to other tasks and may thus affect every major life activity such as foraging, anti-predation, reproduction, mating and social interactions. In natural environments, animals are constantly confronted with multiple signals they should or should not respond to. Cognitive processes, including shared attention to simultaneously present stimuli (or tasks such as foraging and neighbor inspection – both need at the cognitive level perception and processing of cues of prey/food and conspecific individuals), and the associated behaviours are inevitably linked (Bernays 2001, Dukas 2002, 2004). Attention per se can only be indirectly deduced from behavioural and/or neurobiological and physiological measurements (Dukas and Kamil 2000, Bernays 2001, Dukas 2002, 2004). In an ideal case one would integrate these diverse measurements (Dukas 2002, 2004) but this is extremely difficult to accomplish for most study animals. Thus, all previous studies on limited attention, including the pioneering study by Dukas and Kamil (2000) or more recently Griffiths et al. (2004), Purser and Radford (2011) and our own studies (Strodl and Schausberger 2012a, b), relied on behavioural observations alone to conclude on the interrelated cognitive processes. Previous investigations strongly suggested that the ability to behaviourally respond to signals is limited by various cognitive constraints, which are defined as anything that prevents, delays or increases the costs of focusing on a given task (Bernays 2001, Dukas 2002, 2004). Such constraints may be a low learning rate, insufficient perception, or an imperfect long-term and/or working memory. The classical example comes from blue jays simultaneously performing two different tasks (foraging and predator vigilance), limiting their ability to efficiently respond to a peripherally presented predator model (Dukas and Kamil 2000).

The implication of limited attention theory for group-living animals is that, if familiar group members require less attention than unfamiliar ones, assorting with familiar individuals should lead to increased efficiency in other tasks (Dukas 2002, Griffiths et al. 2004, Strodl and Schausberger 2012a, b, Zach et al. 2012). Thus, in light of limited attention theory, social familiarity, i.e. familiarity among conspecific individuals (Alexander 1974), may confer a selective advantage if it allows to switch attention from costly group member assessments or aggressive interactions to other major life activities such as foraging, predator avoidance, parental care or mating (Dukas 2002) and thereby enhances fitness. To date, the only experimental support for limited attention theory in the context of group-living comes from salmonid fish (Griffiths et al. 2004) and predatory mites (Strodl and Schausberger 2012a, b). Griffiths et al. (2004) demonstrated that individuals with a familiar conspecific neighbour responded more quickly to simulated predator attacks and had higher feeding rates than indivi duals with an unfamiliar conspecific neighbor. Likewise, juvenile predatory mites, *Phytoseiulus persimilis*, held in familiar groups responded more quickly to intraguild predator attacks (Strodl and Schausberger 2012a) and foraged more optimally, i.e. needed less prey at similar developmental speed and body size at maturity, than unfamiliar mites (Strodl and Schausberger 2012b). Nevertheless, except for the study by Strodl and Schausberger (2012a), which shows that socially familiar juvenile mites react more quickly to approaching predators and consequently survive longer under predation risk, experimental studies documenting the adaptive significance of social familiarity are lacking.

Here, we examined the adaptive significance of social familiarity in adult females of the group-living, plant- inhabiting predatory mite *P. persimilis*. We hypothesized that social familiarity reduces the cognitive (due to limited attention) and associated behavioural and physiological (due to decreased stress) costs of group-living. Within-groups, familiar mites should be more likely to associate with each other than unfamiliar mites and social familiarity should enhance the mites’ efficiency in foraging and/or reproduction. In the first experiment, we assessed the influence of social familiarity on within-group association behaviour of adult gravid *P. persimilis* females held in mixed groups of familiar and unfamiliar individuals by determining the familiarity status of each individual’s neighbours and the inter-individual distances of familiar and unfamiliar individuals. To assure that the establishment of social familiarity and its possible consequences occur independently of the degree of genetic relatedness (Schausberger 2007), we used females with varying degrees of genetic relatedness. In the second experiment, we assessed the effects of familiarity on general activity, predation, oviposition, inter-individual distances and offspring sex ratio of *P. persimilis* females living in groups consisting of either familiar or unfamiliar individuals. To determine if group size during the sensitive familiarization period affects the familiarization process and its possible consequences later in life, we used females familiarized in small and large groups.

## Methods

### Study species

*Phytoseiulus persimilis* lives in groups (Nachman 1981) and is able to discriminate familiar and unfamiliar conspecific individuals (Schausberger 2004, 2005, 2007, Strodl and Schausberger 2012a, b). Unfamiliar group members require more attention because they are more aggressive than familiar ones (Schausberger 2004, 2007). *Phytoseiulus persimilis* is a highly specialized predator of herbivorous tetranychid mites such as the two-spotted spider mite *Tetranychus urticae*. Spider mites are serious pests of many agricultural crops, patchily distributed on their host- plants and so are the predators that forage, reproduce and develop in the spider mite webbings (Sabelis 1985). These circumstances lead to repeated encounters of the predators, raising opportunities to familiarize (Schausberger 2004, 2005, Strodl and Schausberger 2012b). *Phytoseiulus persimilis* has five life stages: egg, larva, protonymph, deutonymph and adult. The predators are eyeless and use tactile and/or short-distance volatile chemosensory cues on the body surface for conspecific recognition. Regarding familiarization, contact in the larval stage (for cohort familiarization), and briefly after oviposition (for mother offspring familiarization) is crucial (Schausberger 2004, 2005, 2007, Strodl and Schausberger 2012a, b).

### Origin and rearing of experimental animals

Experimental animals were withdrawn from a laboratory-reared population of *P. persimilis*, founded with specimens field-collected in Greece (henceforth termed ‘G’). In experiment 1, we additionally used individuals from a laboratory-reared population founded with specimens obtained from a commercial producer of biocontrol agents (BioHelp, Vienna) (henceforth termed ‘B’). Both populations were held on separate artificial rearing units each consisting of a plastic tile placed on a water-saturated foam cube (13 × 13 cm) in a plastic box (20 × 20 cm) half-filled with water and surrounded by water-saturated tissue paper. The predatory mites were fed mixed stages of two-spotted spider mite, *T. urticae*, maintained on whole common bean plants, *Phaseolus vulgaris*, by adding detached spider mite-infested bean leaves onto arenas in three-day intervals. All rearing arenas and experimental cages were stored at 25 ± 1°C, 60 ± 5% relative humidity and 16:8 h light:dark.

### Arenas used for familiarization and experiments

Arenas used to obtain similarly aged predator eggs and to subsequently generate familiar individuals (henceforth termed oviposition/familiarization arenas), and to assess within group association, foraging and oviposition behaviours (henceforth termed experimental arenas) consisted of single bean leaves placed adaxial surface down on a water-saturated foam cube (50 × 50 mm) in a small plastic box (100 × 100 mm) half-filled with water. Strips of moist tissue paper were folded over the edges of the leaves to prevent the mites from escaping. Before adding the predators, each arena was infested with eight *T. urticae* females for two days to deposit eggs to be used as prey by the predators.

### Influence of familiarity on within-group association behaviour (experiment 1)

Experiment 1 aimed at assessing the influence of familiarity on within-group association behaviour of adult gravid *P. persimilis* females. Each group consisted of two pairs of females. Females of a pair were familiar with each other but unfamiliar to females of the other pair. To generate familiar individuals, three gravid predator females were placed on an oviposition/familiarization arena to oviposit. After two days the females were removed while their eggs (< 20/arena) were left to develop to adulthood. Three to four days after reaching adulthood, the females were mated and ready to be used in experiments. Gravid *P. persimilis* females are easily distinguishable from males because they are about three to four times more voluminous than males.

To distinguish familiar and unfamiliar females, they were marked with watercolour dots on their dorsal shields before the experiment (always same unique colour for familiar females but colours switched between replicates). To start the experiment, two pairs of females (familiar within pairs but unfamiliar between pairs) were placed onto an experimental leaf arena with an accessible area of 40 × 40 mm. Each arena was checked for the position of the females after 0.2, 0.5, 1, 1.5, 2, 3.5, 5, 6.5 and 24 h. Timing of the first observation (at 0.2 h) was based on pre- experimental work aiming to determine the time needed by the mites to adjust to the novel environment. The positions of the females were marked on paper sketches, whose size, shape and surface patterns such as leaf veins corresponded to the leaf arenas. After the experiment, the familiarity status of the 1st (i.e. the closest), 2nd (i.e. the 2nd closest) and 3rd (i.e. the 3rd closest) neighbour of each female was determined and all inter-individual distances were measured.

To determine possible effects of the average degree and homogeneity of genetic relatedness among familiar females on within-group association behaviour, we observed groups of females having either a homogeneous or a heterogeneous genetic background. In groups with homogeneous genetic background all females derived from population ‘G’, while in groups with heterogeneous background the females derived from populations ‘G’ and ‘B’. In the latter case, females used to produce the eggs giving rise to the experimental females were mixed on oviposition/familiarization arenas in ratios 1 ‘G’:2 ‘B’ and 2 ‘G’:1 ‘B’. We used 19 groups with homogeneous and 23 groups with heterogeneous genetic background.

### Influence of familiarity on foraging and oviposition behaviours (experiment 2)

In experiment 2, we assessed the effects of social familiarity and group size during the familiarization process on predation, oviposition, general activity, inter-individual distances and offspring sex ratio of *P. persimilis* females held in groups consisting of either three familiar or three unfamiliar individuals. To generate familiar females, three gravid *P. persimilis* females were randomly chosen from population ‘G’, placed on oviposition/familiarization arenas and allowed to forage and oviposit. After two days the females were removed, and their eggs reduced to either six (small familiarization group) or 17 (large familiarization group) per arena and allowed to develop to adulthood. After approximately one week all *P. persimilis* individuals were adult and mated and gravid females were ready to be used in the experiment. All females used in experiment 2 derived from population ‘G’ and had thus the same degree of genetic relatedness to each other.

Each experimental leaf arena (30 × 30 mm) used in experiment 2 harboured 220 ± 20 eggs of *T. urticae* < 48 h old. To start the experiment, three gravid *P. persimilis* females that were either familiar or unfamiliar to each other were placed onto each leaf arena. Females within familiar triplets derived from the same familiarization arena, whereas each female within unfamiliar triplets derived from a different familiarization arena. Except for social familiarity during the experimental phase, everything else such as pre-experimental rearing and familiarization was the same for individuals of both familiar and unfamiliar triplets. Every 24 h the number of spider mite eggs eaten, eggs laid by the predators, general activity (moving or stationary) and position of the predators on the leaf arena (only for the large familiarization group) were recorded over six consecutive days. The position of the females was marked on leaf-shaped paper sketches, which were then used to measure the inter-individual distances. Predator position was only recorded for females derived from large familiarization groups because this characteristic had already been assessed in experiment 1. Every 48 h the females were transferred to new arenas to provide consistent prey availabilities. Predator eggs were left on arenas and reared until adulthood to determine the sex ratios of offspring produced during each 48 h bouts. Familiar and unfamiliar triplets derived from small familiarization groups were replicated 17 and 14 times, respectively; familiar and unfamiliar triplets derived from large familiarization groups were replicated 14 and 16 times, respectively. To avoid any experimenter’s bias, experiment 2 was performed blind, i.e. the experimenter did not know which groups consisted of familiar and unfamiliar individuals.

### Statistical analyses

All statistical analyses were performed using SPSS 15.0.1 for Windows. In experiment 1, we used separate G-tests for goodness of fit (Sokal and Rohlf 1995) for each observation point and genetic background to compare the observed numbers of 1st neighbours of each female of all arenas being familiar and unfamiliar with the expected numbers (33.3% were expected to be familiar). To compare the familiarity status of 1st, 2nd and 3rd neighbours of each female (number of familiar individuals out of four indivi duals each per arena and observation point) over time and in dependence of genetic background, we used generalized estimating equations (Hardin and Hilbe 2003) (GEE; counts of events; autocorrelation structure between observation points and neighbours, respectively, binomial distribution with logit link function) and post-hoc pairwise comparisons of expected marginal means by Šidák-tests. To assess the effects of familiarity and genetic background on inter- individual distances we used GEE (autocorrelation structure between observation points, normal distribution with identity link) with post hoc pairwise comparison of the expected marginal means by least significant difference (LSD) tests. Due to the fact that individuals within familiar pairs had the same colour, preventing us from tracking single individuals over time, we averaged the inter-individual distances between familiar and unfamiliar females per arena and observation point before analysis. Averages were weighted by 2 (familiar) and 4 (unfamiliar), respectively, according to the number of distances they were calculated from.

In experiment 2, we used separate GEEs (autocorrelation structure between observation days; normal distribution with identity link function except for activity levels, which had a binomial distribution with logit link function) to analyze the effects of familiarity and familiarization group size (both used as between subject variables) and observation day (within subject variable) on daily oviposition and predation rates, offspring sex ratio, and activity levels of mite triplets, and the effects of familiarity and observation day on inter-individual distances in the subset with the large familiarization group size. Predation and oviposition rates and offspring sex ratio were normally distributed (Kolmogorov–Smirnov, p > 0.05). Sex ratio was expressed as the proportion of females among offspring (number of females divided by the total number of offspring). Activity levels were quantified as the number of moving females out of all females per arena and observation point. Inter- individual distances were averaged per arena and the average used for analysis. Non-significant three-way interactions were removed from the model to improve fit of the model.

## Results

### Influence of familiarity on within-group association behaviour (experiment 1)

**Figure 1:**
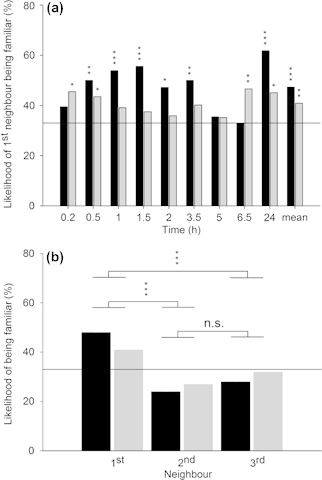
The percentage of 1st neighbours (a) and of 1st, 2nd and 3rd neighbours (b) being familiar within mixed groups of familiar and unfamiliar *P. persimilis* females, with either homo geneous (black bars) or heterogeneous (grey bars) genetic background, over time (a) and across time (b), respectively. Asterisks above bars (a) denote the results of separate G-tests for goodness of fit of the observed numbers of 1st neighbours being familiar and unfamiliar with the expected numbers if random (ratio 1:2) for each observation point and the mean numbers across time. Asterisks above horizontal brackets (b) denote the results of pairwise neighbour comparisons (Šidák tests following GEE) (*p < 0.05, **p < 0.01, ***p < 0.001, n.s. = non-significant). The reference lines represent the expected likelihood of 33% being familiar if random.

The frequency of two neighbouring females being familiar was significantly higher than expected by chance at six of nine observation points in groups with homogeneous genetic background and four of nine observation points in groups with heterogeneous genetic background (Fig. 1a). GEE revealed that the relative frequency of being familiar differed among 1st, 2nd and 3rd neighbours independent of the genetic background and time (Table 1, Fig. 1b). Pairwise comparisons of the estimated marginal means showed that 1st neighbours were more often familiar than 2nd and 3rd neighbours (p < 0.001), whereas the frequencies of 2nd and 3rd neighbours being familiar did not differ (p = 0.214).

**Table 1:** Results of generalized estimating equations (GEE) on the influence of neighbour (1st, 2nd or 3rd) and genetic background (homo- or heterogeneous) on the likelihood of being familiar in mixed groups of familiar and unfamiliar *P. persimilis* females over time.

Source of variation	Wald-χ^2^	DF	p
Neighbour	17.381	2	< 0.001
Background	3.262	1	0.071
Neighbour × Background	1.566	2	0.457
Neighbour × Time	25.460	16	0.062
Background × Time	7.604	8	0.473
Neighbour × Background × Time	26.121	16	0.052

**Figure 2:**
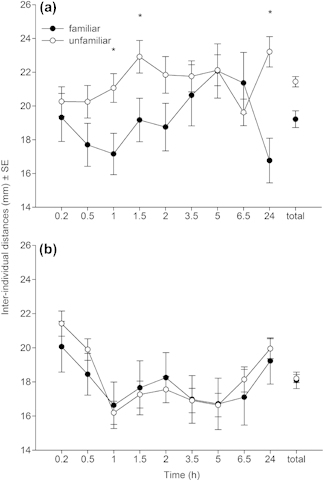
Inter-individual distances (mm; mean ± SE) between familiar and unfamiliar *P. persimilis* females in groups with either homogeneous (a) or heterogeneous (b) genetic background over time (experiment 1). Asterisks above lines denote significant differences (*p < 0.05) between the distances of familiar and unfamiliar females at a given observation point.

Irrespective of the genetic background, the inter-individual distances of familiar and unfamiliar females did not differ across time, but differed at three of nine observation points in the group with homogeneous genetic background (Table 2, Fig. 2). The inter-individual distances were generally shorter in the group with heterogeneous genetic background (Fig. 2b) than in the group with homogeneous genetic background (Fig. 2a). The significant three-way interaction indicates that the distances fluctuated differently over time in dependence of familiarity and genetic background. In the group with heterogeneous genetic background, irrespective of familiarity, the inter-individual distances decreased during the 1st h, stayed at this level until 6.5 h and were back to the initial level after 24 h (Fig. 2b). In contrast, in the group with homogeneous genetic background, the distances between unfamiliar individuals gradually increased from the beginning until 24 h, while those between familiar individuals decreased during the 1st h, afterwards increased until 6.5 h and were the shortest after 24 h (Fig. 2a).

**Table 2:** Results of generalized estimating equations (GEE) on the effects of familiarity and genetic background (homo- or hetero geneous) on inter-individual distances in mixed groups of familiar and unfamiliar *P. persimilis* females over time.

Source of variation	Wald-χ^2^	DF	p
Familiarity	2.397	1	0.122
Background	6.831	1	0.009
Background × Familiarity	1.385	1	0.239
Familiarity × Time	3.029	8	0.189
Background × Time	55.579	8	< 0.001
Familiarity × Background × Time	19.074	8	0.014

### Influence of familiarity on foraging and oviposition behaviours (experiment 2)

**Figure 3:**
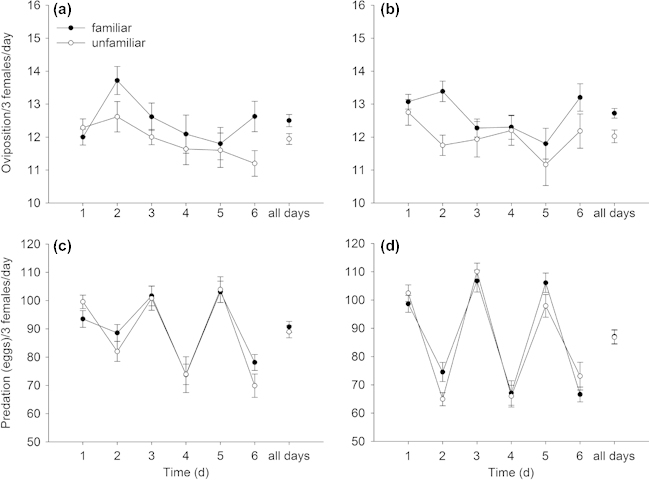
Mean (± SE) daily oviposition rate (a, b) and daily predation rate (c, d) of triplets of either familiar or unfamiliar *P. persimilis* females over six days. Group size during the familiarization phase was either 17 (a, c) or 6 (b, d).

**Figure 4:**
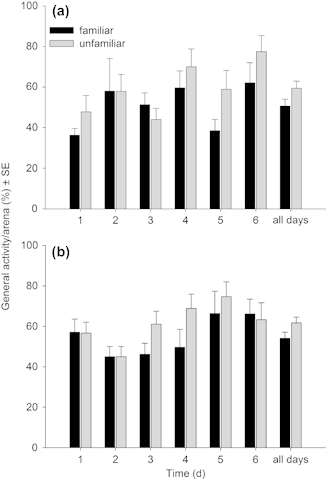
Mean daily activity (% females moving ± SE) within triplets of either familiar or unfamiliar *P. persimilis* females over six days. Group size during the familiarization phase was either 17 (a) or 6 (b).

**Figure 5:**
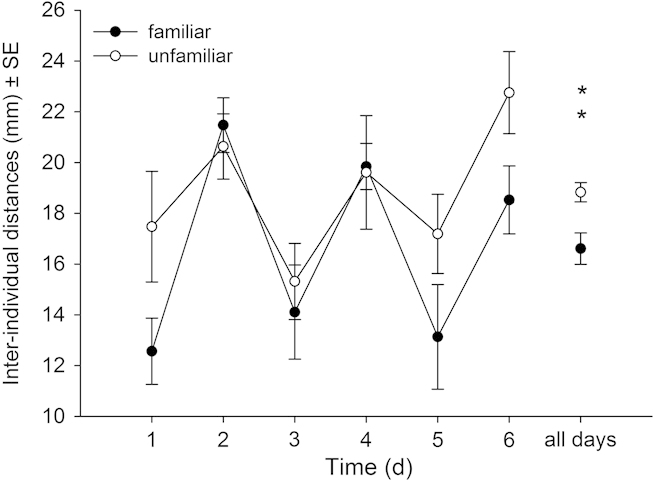
Inter-individual distances (mm; mean ± SE) within triplets of either familiar or unfamiliar *P. persimilis* females over six days (experiment 2). Group size during the familiarization phase was 17. Asterisks above symbols for all days refer to the results of GEE for the main effect of familiarity (**p < 0.01).

Irrespective of familiarization group size (6 or 17), females within familiar groups had higher oviposition rates (Table 3, Fig. 3a–b) and lower general activity levels (Table 3, Fig. 4a–b) than females within unfamiliar groups. The predation rates did not differ between females of familiar and unfamiliar groups (Table 3, Fig. 3c–d). General activity levels of females familiarized in small and large groups increased similarly over time but the former were overall less active than the latter (Fig. 4a–b). The inter- individual distances between females of familiar groups were significantly lower than those between females of unfamiliar groups. The distances between familiar females decreased while those between unfamiliar females increased over time (Table 3, Fig. 5). The cyclic two-day fluctuations – increasing inter-individual distances with decreasing prey density from the 1st to the 2nd day on a given arena – were caused by transferring the predators to new arenas every other day. Offspring sex ratio (female proportion) was not influenced by familiarity (mean ± SE: familiar 0.71 ± 0.03 vs unfamiliar 0.74 ± 0.02; GEE: Wald-χ_1_^2^= 0.746, p = 0.388) and familiarization group size (0.74 ± 0.03 for group size 6 vs 0.70 ± 0.03 for group size 17; Wald-χ_1_^2^= 1.074, p = 0.300) but increased marginally significantly over time (Wald-χ_2_^2^= 5.804, p = 0.085).

**Table 3:** Results of generalized estimating equations (GEE) for the influence of familiarity and group size during familiarization (6 or 17) on oviposition rate, predation rate, and general activity, and for the influence of familiarity on inter-individual distances (familiar ization group size 17) of triplets of *P. persimilis* females over time.

Parameter	Source of variation	Wald-χ^2^	DF	p
Oviposition	Familiarity	9.439	1	0.002
	Group size	0.585	1	0.444
	Group size × Familiarity	0.048	1	0.826
	Time × Familiarity	13.058	5	0.023
	Time × Group size	13.919	5	0.016
Predation	Familiarity	0.413	1	0.520
	Group size	2.839	1	0.092
	Group size × Familiarity	0.001	1	0.988
	Time × Familiarity	10.094	5	0.073
	Time × Group size	30.155	5	< 0.001
Activity	Familiarity	11.681	1	0.001
	Group size	4.360	1	0.037
	Group size × Familiarity	0.368	1	0.605
	Time × Familiarity	4.574	5	0.470
	Time × Group size	16.592	5	0.005
Distances	Familiarity	9.383	1	0.002
	Time × Familiarity	89.936	5	< 0.001

## Discussion

Social familiarity had a decisive impact on within-group association behaviour and reproduction of the predatory mite *Phytoseiulus persimilis*. Females living in mixed groups of familiar and unfamiliar females preferentially associated with familiar females. This behaviour was more apparent in groups with homogeneous genetic background than in groups with heterogeneous genetic background. Females living in groups of only familiar females had higher oviposition rates, similar predation rates, lower activity levels and shorter inter-individual distances than females living in groups of only unfamiliar females. Limited attention theory postulates that in multiple signal environments attention has to be shared among signals, with familiar signals drawing off less attention than unfamiliar signals (Dukas 2002). Thus, our findings are consistent with the idea that familiar pre datory mite females preferentially assort with each other because a familiar social neighbourhood draws off less attention and allows direction of more attention and energy to fitness enhancing activities such as oviposition.

Our study documents and links the adaptive significance of social familiarity to relaxation of limited attention (Dukas 2002), reducing the physiological costs of infor mation gathering and processing in a group-living animal. In general, physiological tradeoffs may occur for various reasons and behavioural observations are only indirect indicators of attention. Nevertheless, in our experiments social familiarity, a cognitive trait, was the only manipulated factor, everything else was kept the same. Moreover, the assumption of the tight linkage between attention-related cognitive processes, fitness and behavioural traits is backed up by the recent finding that social familiarity enhances vigilance, measured in reaction time, and survival of *P. persimilis* under the risk of predation (Strodl and Schausberger 2012a). Reaction time is a most commonly used behavioural indicator of attention (Rowe 1999, Griffiths et al. 2004). Various studies suggested beneficial effects of social familiarity in group-living species con cerning foraging, anti-predator behaviour and agonistic inter actions (Chivers et al. 1995, Höjesjö et al. 1998, Utne-Palm and Hart 2000, Strodl and Schausberger 2012a, b). However, no previous study measured the effects of social familiarity on the reproductive success of group-living animals. For example, minnows in familiar shoals had higher food intake rates than minnows in unfamiliar shoals (Chivers et al. 1995). However, higher feeding rates do not necessarily convert into higher reproduction (Illius et al. 2002). In experiment 2, females living in familiar groups had higher oviposition rates than females living in unfamiliar groups. Classical optimal foraging theories predict that selection favours decision mechanisms, which optimize energy gain by maximizing food intake rate and/or minimizing handling and/or searching times (Giraldeau 2008). Consequently, in terms of limited attention, familiarity could have increased foraging success of familiar individuals (Griffiths et al. 2004). However, the 24 h predation rates of *P. persimilis* did not differ and possible differences in handling and searching times were negligible due to the high prey density. There was also no indication of partial vs complete consumption of prey eggs between-groups, which could have resulted in differences in energy extracted per prey item. Moreover, such differences could have been balanced by shorter handling times, reducing energy spent per item, and could have been compensated for by increased numbers of consumed eggs. We therefore argue that the observed lower general activity level of familiar females is the proximate explanation for their higher reproduction. Lower general activity is an indicator of decreased restlessness, which, at the physiological level, reflects less stress (Lincoln et al. 1998 for a definition of stress, Takeda et al. 2003), and, at the cognitive level, a reduced need to explore the immediate social surrounding, leading to lower energy expenditure for neighbour inspection and assessment (Lima 1998, Dukas 2002) and leaving more energy for egg production. In general, differences in energy expenditure may also be due to other constraints than behavioural performance such as oxygen availability, temperature, diurnal activity, life stage and phase, etc. However, these other constraints can be dismissed as potential explanations in our study because, except for social familiarity during the experiment, everything else, such as the conditions during pre-experimental rearing or life stage and phase, was the same for individuals of familiar and unfamiliar groups. An alternative behavioural inter pretation is that lower activity indicates a lower dispersal propensity of familiar individuals, leading to higher energy expenditure and reduced egg production in unfamiliar individuals. However, this explanation can be dismissed because mites held in familiar groups have a similar or greater propensity to disperse than mites held in unfamiliar groups (Zach et al. 2012). Thus, the activity levels measured in experiment 2 are only indicative of within group behaviour but not dispersal or between group movements.

Depending on the ecological context and the life-stages involved, *P. persimilis* is able to use at least three different perceptual mechanisms, enabling them to discriminate familiar from unfamiliar conspecifics or kin from non- kin: recognition via prior association, phenotype matching and self-referent phenotype matching (Schausberger 2004, 2005, 2007, Strodl and Schausberger 2012a, b). In the current experiments, *P. persimilis* seems to have used individual recognition of genetically determined tactile or short distance volatile, recipient-borne chemosensory cues learned through prior association. The response of females living in groups with homogeneous and hetero geneous genetic background did not fundamentally differ, implying that close genetic relatedness per se was not a precondition to familiarize and later discriminate familiar and unfamiliar individuals. Some fishes may even discriminate between familiar and unfamiliar heterospecific individuals (Ward et al. 2003, Valero et al. 2009). However, statistical analyses revealed sophisticated subtle differences in inter-individual distances (indicated by the three-way interaction). Familiar females with homogeneous genetic background were closer together than unfamiliar females at the beginning of the experiment (until 1.5 h) but not during the subsequent 4 h. This pattern might indicate that familiar females were more strongly attracted to each other in the initial phase of the experiment when they were heavily stressed and disordered due to having been transferred into a novel environment. After acclimatization, the predators resumed foraging and searching for optimal oviposition sites, activities expected to increase the inter-individual distances over time because food availability decreases. Nonetheless, the shortest distances and highest likelihood of familiar females being 1st neighbours occurred at the last observation point (after 24 h) when prey density was low but predator egg density high. *Phytoseiulus persimilis* females are well able to adjust patch-leaving to own and progeny prey needs (Vanas et al. 2006), to manipulate hatching asynchrony to reduce the risk of sibling cannibalism (Schausberger and Hoffmann 2008), and adjust egg placement to ensure that own offspring imprint on kin (Schausberger 2005). Therefore, it is possible that closer association after 24 h was additionally determined by the presence of eggs, i.e. familiar females depositing their eggs closer together, thereby amplifying the effect of familiarity on inter-individual distances. We did not observe this phenomenon in groups of females having a heterogeneous genetic background. In the heterogeneous group, familiarity did not have any effect on inter-individual distances whereas in the homogeneous group it had a significant effect at three observation points. These comparisons indicate that learning and responding to individual cues is perceptually more challenging in a genetically heterogeneous than homogeneous group and may consequently compromise precision in individual recognition. Furthermore, in experiment 2, we observed a more pronounced effect of familiarity on inter-individual distances than in experiment 1. Recognition was probably more precise in experiment 2 because individuals were perceptually less challenged due to lower within-group label variability.

We did not show that females discriminate between different individuals having exactly the same familiarity status, which is experimentally extremely difficult if not impossible to achieve (Thom and Hurst 2004), but we argue that individual recognition, which is known from various animals (Halpin 1980, Bonadonna and Nevitt 2004, Schausberger 2007, Sheehan and Tibbetts 2008), is nonetheless the most likely and plausible recognition mechanism. In experiment 2, group size during familiarization did not affect discrimination ability: females reared in groups of 17 were equally able to discriminate between familiar and unfamiliar individuals as were females reared in groups of 6. This outcome suggests that each female learned and memorized up to 16 individual labels. An alternative explanation to individual recognition is marking of group members leading to a shared phenotypic cue of familiar individuals. During familiarization each individual could have deposited a unique chemical marker on each encountered individual, allowing to later re-recognize this marked individual, or the markers of all group members intermingled and created a unique group-specific mixture that was learned and later re-recognized by each group member. Con- and/or hetero-specific marking is known from various arthropods (Breed et al. 1992, Ivy et al. 2005, Yew et al. 2009) but is unlikely in our experiments for the following reasons: 1) individuals touch each other with the 1st pair of legs for recognition but no glands are known on these legs that could possibly produce marking pheromones (Jagers op Akkerhuis et al. 1985); 2) this mechanism would require repeated marking because the mites moulted three times during the familiarization phase; 3) if every conspecific individual encountered is marked, the effect of familiarity should have vanished over time in the course of the experiment but the opposite was the case – the effect of familiarity was rather strengthened than weakened over time; 4) in experiment 2, every individual would have received up to 16 different markers likely resulting in complex mixtures and possibly masking each other, rendering re-recognition of unique markers extremely difficult.

In summary, our study suggests that social familiarity may reduce the costs of cognitively and physiologically challenging group-living. The observed adaptive significance of social familiarity linked to relaxed limited attention suggests that limited attention may be an important ubiquitous driver of the evolution of the ability to discriminate familiar and unfamiliar individuals in group-living animals. We expect further implications of social familiarity to major life processes of both adult and immature life-stages, not only in reproduction but also in anti-predator behaviours against heterospecific predators (Griffiths et al. 2004, Strodl and Schausberger 2012a), agonistic conspecific interactions (Utne-Palm and Hart 2000, Schausberger 2004, 2007), or juvenile growth and survival (Gerlach et al. 2007, Strodl and Schausberger 2012b). The idea that the effects of social familiarity should cascade to the population level and translate into higher population productivity due to optimized patch exploitation (Vanas et al. 2006, Zach et al. 2012) opens a promising avenue of future research.
